# Characterizing the Access of Cholinergic Antagonists to Efferent Synapses in the Inner Ear

**DOI:** 10.3389/fnins.2021.754585

**Published:** 2021-12-14

**Authors:** Choongheon Lee, Anjali K. Sinha, Kenneth Henry, Anqi W. Walbaum, Peter A. Crooks, Joseph C. Holt

**Affiliations:** ^1^Department of Otolaryngology, University of Rochester, Rochester, NY, United States; ^2^Department of Neuroscience, University of Rochester, Rochester, NY, United States; ^3^Department of Pharmaceutical Sciences, College of Pharmacy, University of Arkansas for Medical Sciences, Little Rock, AR, United States; ^4^Department of Pharmacology & Physiology, University of Rochester, Rochester, NY, United States

**Keywords:** vestibular efferents, auditory efferents, nicotinic, muscarinic, mouse, DPOAE

## Abstract

Stimulation of cholinergic efferent neurons innervating the inner ear has profound, well-characterized effects on vestibular and auditory physiology, after activating distinct ACh receptors (AChRs) on afferents and hair cells in peripheral endorgans. Efferent-mediated fast and slow excitation of vestibular afferents are mediated by α4β2*-containing nicotinic AChRs (nAChRs) and muscarinic AChRs (mAChRs), respectively. On the auditory side, efferent-mediated suppression of distortion product otoacoustic emissions (DPOAEs) is mediated by α9α10nAChRs. Previous characterization of these synaptic mechanisms utilized cholinergic drugs, that when systemically administered, also reach the CNS, which may limit their utility in probing efferent function without also considering central effects. Use of peripherally-acting cholinergic drugs with local application strategies may be useful, but this approach has remained relatively unexplored. Using multiple administration routes, we performed a combination of vestibular afferent and DPOAE recordings during efferent stimulation in mouse and turtle to determine whether charged mAChR or α9α10nAChR antagonists, with little CNS entry, can still engage efferent synaptic targets in the inner ear. The charged mAChR antagonists glycopyrrolate and methscopolamine blocked efferent-mediated slow excitation of mouse vestibular afferents following intraperitoneal, middle ear, or direct perilymphatic administration. Both mAChR antagonists were effective when delivered to the middle ear, contralateral to the side of afferent recordings, suggesting they gain vascular access after first entering the perilymphatic compartment. In contrast, charged α9α10nAChR antagonists blocked efferent-mediated suppression of DPOAEs only upon direct perilymphatic application, but failed to reach efferent synapses when systemically administered. These data show that efferent mechanisms are viable targets for further characterizing drug access in the inner ear.

## Introduction

Efferent innervation of the mammalian inner ear begins as bilateral clusters of predominantly cholinergic neurons in several distinct nuclei within the pontomedullary regions of the brainstem. The cell bodies of vestibular and auditory efferent neurons are found in group e and the superior olivary complex, respectively (Warr, 1975; Goldberg and Fernández, 1980; Leijon and Magnusson, 2014). They give rise to axons that collect in the eighth cranial nerve on both sides and travel to the inner ear to innervate hair cells and/or primary afferents in the vestibular endorgans and cochlea (Guinan, 2006; Maison et al., 2013; Holt, 2020; Poppi et al., 2020). In both efferent systems, contralateral efferent neurons, destined to innervate the ipsilateral ear, cross the midline just below the floor of the fourth ventricle. This anatomical confluence has provided a convenient place to electrically stimulate both vestibular and auditory efferent neurons in studies that seek to characterize peripheral efferent synaptic mechanisms and how they impact inner ear function (Goldberg and Fernández, 1980; Sridhar et al., 1995; Maison et al., 2007; Schneider et al., 2021). Many inner ear efferent neurotransmitters have been identified (Guth et al., 1998; Holt et al., 2011; Sewell, 2011; Kitcher et al., 2021), but much of the pharmacology regarding electrical stimulation of inner ear efferents has demonstrated a major role for acetylcholine (ACh). Targeting these cholinergic efferent mechanisms in the inner ear are not only critical to understanding their roles in vestibular and auditory behaviors, but also offer an opportunity to characterize how different cholinergic agents access the intact inner ear.

Electrical stimulation of vestibular efferent neurons in mammals alters the excitability of primary vestibular afferents along several time scales indicating roles for multiple postsynaptic mechanisms (Goldberg and Fernández, 1980; McCue and Guinan, 1994; Marlinski et al., 2004; Schneider et al., 2021). Recent pharmacological evidence in mice has identified at least three distinct cholinergic mechanisms. Efferent-mediated slow excitation requires activation of afferent muscarinic ACh receptors (mAChRs) while efferent-mediated fast excitation depends on activation of afferent α4β2*-containing nicotinic AChRs (nAChRs) (Ramakrishna et al., 2020; Schneider et al., 2021). Efferent-mediated inhibition of vestibular afferents is thought to proceed through the sequential activation of α9α10nAChRs and SK2 potassium channels in type II vestibular hair cells (Poppi et al., 2018, 2020; Yu et al., 2020). While patch clamp recordings demonstrate that α9α10nAChRs and SK2 are widely expressed in type II hair cells, direct observations of efferent-mediated inhibition of mammalian vestibular afferents are infrequent (Goldberg and Fernández, 1980; Marlinski et al., 2004; Schneider et al., 2021). Efferent-mediated inhibition is likely obscured by contemporaneous efferent-mediated afferent excitation (Holt et al., 2015). As such, selective pharmacological blockade of the excitatory components, particularly fast excitation, is needed to unmask the underlying efferent-mediated inhibition, before confirming that α9α10nAChRs and SK2 are involved. Fortunately, an alternative and reliable source of efferent-mediated activation of the same inhibitory mechanism in the inner ear can be found on the auditory side. Electrical stimulation of medial olivocochlear efferent neurons also activates α9α10nAChRs and SK2 to hyperpolarize outer hair cells (OHCs). This hyperpolarization results in a robust suppression of distortion product otoacoustic emissions (DPOAEs) and compound action potentials (CAPs) (Sridhar et al., 1995; Maison et al., 2007), which could be reliably monitored to determine whether selective α9α10nAChR antagonists access the inner ear.

Many pharmacological studies characterizing the underlying cholinergic efferent receptor mechanisms in the mammalian inner ear have been carried out in anesthetized or reduced preparations (Sridhar et al., 1995; Maison et al., 2007; Poppi et al., 2018; Ramakrishna et al., 2020; Schneider et al., 2021), while insights into vestibular and auditory efferent function in behaving animals have been primarily performed in transgenic animals missing key efferent synaptic mechanisms (Lauer and May, 2011; May et al., 2011; Luebke et al., 2014; Hübner et al., 2015, 2017; Terreros et al., 2016; Clause et al., 2017; Morley et al., 2017; Tu et al., 2017; Jones et al., 2018; Wang et al., 2021). The further incorporation of pharmacological tools in probing efferent function in behaving animal models could be used to corroborate those observations in transgenic animals, provided that the drugs used are selective and their application can be restricted to the inner ear while limiting CNS entry. Drug entry into the inner ear has been modeled in part on entry of the same drugs into the CNS given some similarities between the blood–brain barrier (BBB) and the blood-labyrinth barrier (BLB) (Salt and Hirose, 2018; Salt and Plontke, 2018; Nyberg et al., 2019; Walia et al., 2021), although the BLB is thought to be more permeable than the BBB. To date, systemically-administered drugs used to block inner ear efferent mechanisms in mammals, including atropine, scopolamine, dihydro-β-erythroidine, and strychnine (Maison et al., 2007; Schneider et al., 2021), are all small-molecular weight tertiary amines that also cross the BBB. Alternatively, some selective cholinergic antagonists have charged quaternary ammonium heads which can significantly limit their access into the CNS, but details about whether they can access the inner ear are unknown. Previous experiments using the ionic tracer trimethylphenylammonium (TMPA) or the biscationic AMPA receptor blockers IEM1460 and IEM1925 reveal that some positively-charged substances can enter the ear upon systemic administration (Mikulec et al., 2009; Walia et al., 2021). While the IEM compounds retain key physiochemical properties that favor CNS entry, TMPA does not (Daina et al., 2017). This begs the question as to whether charged cholinergic drugs, particularly those that exhibit little to no BBB permeability, can also travel to the inner ear.

In this study, using different drug administration routes, we utilized a combination of vestibular afferent and DPOAE recordings during electrical stimulation of vestibular and auditory efferent neurons, before and after the administration of charged mAChR and α9α10nAChR antagonists with limited BBB permeability. Our pharmacological data reveal that charged mAChR antagonists access the inner ear independent of the administration route and can move from one ear to the other, while the charged α9α10nAChR antagonists appear effective only when injected directly into the perilymphatic compartment. Charge, structure, and size of the drug molecules likely contribute to their relative access among the various compartments.

## Materials and Methods

### Animals

All animal procedures were performed in accordance with NIH’s Guide for the Care and Use of Laboratory Animals, and approved by the University Committee for Animal Resources (UCAR) at the University of Rochester Medical Center (URMC). Mice: Both sexes of C57BL/6 mice (Jackson Laboratory), weighing 20–30 g, and aged 49–180 days were housed in a one-way room with a standard 12-h light:dark cycle and free access to food and water. Turtle: Both sexes of Red-eared slider turtles (*Trachemys scripta elegans*, 100–500 g, ∼7–18 cm carapace length) were obtained from Cyr Biology Company (Ponchatoula, LA, United States). They were group-housed in large polycarbonate tanks with running water, basking structures, heat lamps, and 12-h light:dark cycle.

#### Mouse Preparation

Details of our mouse preparation were previously published (Schneider et al., 2021). Briefly, mice were deeply anesthetized with (IP) urethane (1.6 g/kg) and xylazine (20 mg/kg). Heart rate was continuously monitored using a 3-lead EKG and body temperature (∼36.5-37.5°C) was maintained using a homeothermic monitoring system (Harvard Apparatus). A tracheostomy was performed for intubation and mechanical ventilation at a rate of 100 bpm (model 683, Harvard Apparatus). After the head was secured in a stereotaxic frame (Stoelting), a posterior craniotomy and cerebellar aspiration were performed to expose cranial nerve VIII on the right side just before it enters the otic capsule and/or the floor of the 4th ventricle.

#### Turtle Preparation

Details of the turtle preparation were published previously (Holt et al., 2006, 2015, 2017). Briefly, turtles were deeply anesthetized with Euthasol (40–100 mg/kg). Once areflexic, they were decapitated and the head was split along the sagittal axis. The left half was immersed in an oxygenated artificial perilymph (AP) solution (in mM): 105 NaCl, 4 KCl, 0.8 MgCl_2_, 2 CaCl_2_, 25 NaHCO_3_, 2 Na-pyruvate, 0.5 glutamine, 10 glucose, pH 7.2–7.3 during continuous bubbling with 95% O_2_/5% CO_2_. Much of the remaining brain in the left half-head was removed and a small opening made in the temporal bone exposed the posterior ampullary nerve with its two branches to the crista epithelium, whereby connective tissues on the nerve’s surface were carefully peeled back with a fine tungsten hook. The half-head preparation, anchored into a plastic recording chamber using cyanoacrylate, was moved to the recording rig whereby the exposed posterior ampullary nerve was continuously supplied with oxygenated AP.

### Afferent Recordings

Sharp microelectrodes, with impedances of 40–120 MΩ, were pulled from borosilicate glass tubing (BF150-86-10, Sutter Instrument), filled with 3 M KCl, and inserted into an electrode sleeve connected to a single axis motorized micromanipulator (IVM, Scientifica). After connecting to a preamplifier headstage (Biomedical Engineering, Thornwood, NY, United States), microelectrodes were lowered into the superior division of nerve VIII in mouse or the posterior crista nerve of turtle to record extracellular spike activity from spontaneously-discharging vestibular afferents. Afferent signals were low-pass filtered (1 kHz, four-pole Bessel; Wavetek), sampled at 10 kHz, and recorded using in-house acquisition scripts in Spike2 (Cambridge Electronic Design) on a PC with a micro1401 interface. Spike2 data files, exported as general text files, were processed with custom macros in IgorPro 8.02 (WaveMetrics). Afferent discharge in mice and turtle was classified according to CV*, a normalized measurement of discharge regularity (Brichta and Goldberg, 2000a; Schneider et al., 2021). Mouse afferents were classified as regularly-discharging when CV* < 0.1, while afferents with CV* > 0.1 were classified as irregularly-discharging. A total of 58 mice and 16 turtles were used for afferent recordings in this study.

### Distortion Product Otoacoustic Emissions

Stimulus components F1 (10 KHz) and F2 (12 KHz) were presented independently using separate Etymotic ER2 earphones coupled to the ear canal through a 3-mm tip and an ER10-B+ low-noise microphone system. F1 and F2 were generated with 16-bit resolution on two analog output channels of a data acquisition card (PCIe-6251; National Instruments) and scaled to the desired level with two programmable attenuators (PA5; Tucker Davis Technologies). F1 level ranged 40–70 dB SPL with F2 always 10 dB < F1. Two headphone drivers (−27 dB gain; HB7; Tucker Davis Technologies) powered the earphones. Microphone output was amplified (40 dB gain; ER10-B+) and sampled using the same data acquisition card. Sampling frequencies of analog I/O were 50 kHz. DPOAE recordings were controlled with custom programs written in MATLAB (The MathWorks). F1/F2 stimuli were presented every 2.3 s (2.05 s w/0.025-s cosine-squared onset/offset ramps and 0.25 s of silence). DPOAE amplitudes were measured during the unramped period by first dividing the sampled microphone input into four 0.5 s segments and then averaged to reduce noise levels. DPOAE amplitude (dB SPL) and noise level were calculated from the Fourier transform of the average response at 2*F1 – F2. Noise level was estimated from subtracting the average responses of segments 1 and 3 from segments two and four. The stimulus frequencies and levels used in this study are typically associated with a 5–15 dB suppression of DPOAEs during efferent stimulation and thought to target some of the peak efferent innervation densities along the outer hair cell region (Maison et al., 2003, 2007). A total of 44 mice were used for DPOAE recordings in this study.

### Efferent Stimulation

In mice, a platinum-iridium rake of four separate electrodes was lowered into the floor of the 4th ventricle along the midline and just caudal to the facial colliculi (Schneider et al., 2021). At this location, the same electrode configuration can stimulate both medial olivocochlear efferents and contralateral vestibular efferents crossing over to innervate the contralateral ear. To stimulate efferent neurons in turtle, the tip of one Teflon-coated silver/silver chloride wire (AG10T; Medwire, Mt. Vernon, NY, United States) was placed on the cross-bridge, a small nerve bundle of predominantly efferent fibers connecting the anterior and posterior divisions of the VIIIth nerve (Fayyazuddin et al., 1991), while a second electrode was placed on nearby bone. For both preparations, efferent stimuli were produced using laboratory-designed Spike2 scripts on a PC where TTL pulses from a digital-output port of a micro1401 interface (Cambridge Electronic Design) triggered a stimulus isolator (model A360; World Precision Instruments, Sarasota, FL, United States) to deliver current pulses to efferent electrodes. In all preparations, electrical stimuli consisted of trains of 100–150 μs constant-current shock pulses delivered from the stimulus isolator to any single electrode pairs. We varied the amplitude of shock pulses to determine the threshold (T, 20–50 μA) and maxima (75–800 μA) that elicited robust suppression of DPOAEs or afferent responses without antidromic activation. Shock trains consisted of 20 shocks at 200/s in turtle, 333 shocks/s for 5 s for mouse vestibular afferent recordings, and 200 shocks/s for 70 s for mouse DPOAE recordings. Inter-trial intervals between successive shock trains were 3–5, 60–75, and 250–350 s for the three preparations, respectively. These intervals were needed for efferent-mediated responses to return to baseline values before the arrival of the next shock train.

Efferent shock artifacts were canceled off-line after computing an average artifact and subtracting it from corresponding records. Mean afferent or DPOAE responses to efferent stimulation were calculated by averaging 3–25 trials during each experimental condition. Shock train start was always set to *t* = 0 and spike times or DPOAE amplitude measurements were specified for each trial starting at 0.5–40 seconds before the efferent shock train and ending at 0.5–40 seconds before the next efferent shock train. Responses to successive efferent shock trains were also displayed as continuous response graphs to capture the succession of sequential shock trains and reveal the serial effects of a particular treatment. As a function of efferent stimulation paradigms and the resulting kinetics of different efferent-mediated responses, response amplitudes were measured from different time segments in each species in accordance with previously published work (Brichta and Goldberg, 2000b; Maison et al., 2007; Holt et al., 2015; Schneider et al., 2021). For turtle vestibular afferent recordings, the mean amplitude of efferent-mediated inhibition or excitation was calculated from the first 100-ms segment of the average response histogram immediately following the efferent shock train. In mice, mean peak amplitude of efferent-mediated fast excitation was tabulated from the first 500-ms segment of the average response histogram starting at *t* = 0 s. The mean peak amplitude of efferent-mediated slow excitation was computed from a 1-s segment at *t* = 6–7 s, a region typically including the maximum efferent-mediated slow excitation but excluding any efferent-mediated fast excitation. Finally, in the mice DPOAE recordings, measurements of peak efferent-mediated DPOAE suppression were taken from the minimal DPOAE amplitude observed during the first 10 s of the efferent stimulus for each average response under control conditions. Subsequent measures of efferent-mediated suppression of DPOAE amplitude during drug administration were measured using the same time point as the respective control records. To avoid contamination by efferent-mediated suppression, the mean amplitude of the efferent-mediated slow enhancement was calculated from the range of DPOAE values taken at 140–155 s after shock-train onset. All reported mean response amplitudes include a subtraction of mean prestimulus background discharge rates or DPOAE amplitudes taken from the 0.5–10 s of the prestimulus period (*t* = −40 to 0).

### Drug Administration

Afferent or DPOAE responses to efferent stimulation were acquired before, during, and after the administration of pharmacological agents. In turtle, drugs were prepared in turtle Ringers and administered directly to the neuroepithelium using a gravity-fed, multibarrel pipette. In mice, administration routes included intraperitoneal (IP) injection, delivery into the middle ear space using an intrabulla (IB) approach, or direct perilymphatic delivery via an intracanal (IC) approach through the posterior canal. For the IB approach, we first made a small incision behind the right pinna and then retracted the underlying muscles to identify the posterior bulla. We made a small opening in the otic bulla using a 30-G needle where we inserted the pulled 50–200 μm tip of a plastic 1-ml syringe and then sealed with cyanoacrylate glue. We elected to use an IB route over an intratympanic route as it permitted a bottom-up approach to completely fill the middle ear and submerge the round/oval window without having to contend with residual air pockets that could alter drug movement into the inner ear. The IB route also avoided damage to the tympanic membrane, associated middle ear ossicles, and linkage to the oval window.

The IC approach to access the mouse inner ear has been described (Suzuki et al., 2017; Isgrig and Chien, 2018; Talaei et al., 2019). In short, a postauricular incision behind the ear was made with a micro-scissors and the muscles underlying the temporal bone were separated and retracted, exposing the bony wall of the posterior semicircular canal. In preparation for fenestration of the bony surface, the mucosa was removed and the area was dried with a soft cotton tip. A small area on the posterior semicircular canal was fenestrated (∼150 to 200 μm diameter) with a myringotomy blade (Beaver-Visitec). The perfused solutions for IC administration were prepared in mouse artificial perilymph (in mM: 150 NaCl, 4 KCl, 8 Na2HPO4, 2 NaH2PO4, 1.5 CaCl2, 1 MgCl2, and 10 glucose; pH 7.4). The selected solution was loaded into a 10-μl gastight Hamilton syringe that was connected to a customized polypropylene tube (OD ∼100–120 μm). The distal end of the polypropylene tube was inserted and sealed into the posterior canal with a thin layer of cyanoacrylate glue (Permabond) to prevent leakage of perilymph. The sealed surface was monitored for more than 15 min to confirm that there was no obvious fluid leakage. Typically, a total volume of 1–2 μL was manually delivered into the perilymph over ∼30–60 s at an approximate perfusion rate of ∼33 nL/s. In some cases, multiple IC injections were given in the same animal.

### Drugs Used

The neuromuscular blocker *d*-tubocurarine (dTC, 0.625 mg/kg, IP) was used to suppress muscle contractions occasionally seen with brainstem stimulation. Experimental drugs used in this study included the mAChR antagonists glycopyrrolate (GLY) and methscopolamine (MSC) as well as the α9α10nAChR antagonists strychnine (STR), Cmpd7a (aka ZZ1-61c), Cmpd10c (aka GZ556A), and Cmpd11e (aka ZZ204G). IP doses were as follows: (1) A dose of 2 mg/kg for GLY and MSC was selected in order to compare their effectiveness in this preparation to previously characterized mAChR antagonists (i.e., atropine and scopolamine) (Schneider et al., 2021); (2) The dose for STR (6 mg/kg) was based on previous cochlear efferent studies (Maison et al., 2007); (3) Initial doses for Cmpd7a (1–38 mg/kg), Cmpd10c (2.5–49 mg/kg), and Cmpd11e (2.5–5 mg/kg) in mice were chosen based on previous pharmacological characterization in rodents (Holtman et al., 2011; Wala et al., 2012), while higher doses were sought when lower doses failed to produce an effect; and (4) The concentration range for Cmpds 7a, 10c, and 11e (0.01–2 μM) used in turtle afferent recordings were determined from previous pharmacological characterization in *Xenopus* oocytes (Zheng et al., 2011). IB delivery of glycopyrrolate and methscopolamine typically used ∼30 μL of a 0.2 mg/ml stock for a final concentration of ∼0.5 mM for either drug. IC delivery of glycopyrrolate, Cmpd7a, Cmpd10c, Cmpd11e typically used a 1–2 μL injection volume with drug concentrations ranging from 0.3 to 5 mM. Source of drugs used in this study: Glycopyrrolate, methscopolamine (URMC pharmacy or Sigma); strychnine, dTC (Sigma); Cmpds7a (ZZ1-61c), 10c (GZ556A), and 11e (ZZ204G) were synthesized by the Crooks Lab according to Zhang et al. (2008).

### Statistical Procedures

The effects of different pharmacological treatments on efferent-mediated changes in afferent discharge rate or DPOAE response amplitudes were assessed using a paired *t*-test. A one-way ANOVA was used to compare block times among different drug administration routes. One-sample *t*-test was used to evaluate if means differed from zero. All statistical analyses were done in Graph Pad-Prism (GraphPad). Values, expressed as means ± SEM, and outcome parameters including *p*-values, *F*-statistics, *t*-statistics, and effect sizes (Cohen’s *d*) are reported in the text and/or figures. For paired *t*-tests, Cohen’s *d* (*d*) was tabulated using the equation $d=t÷\sqrt{N}$ where *t* is the *t*-statistic and *N* is the sample size.

## Results

**FIGURE 1:**
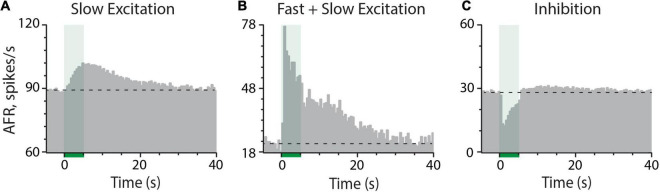
Electrical stimulation of vestibular efferent neurons can elicit three distinct effects on afferent discharge including **(A)** slow excitation, **(B)** combined fast and slow excitation, and **(C)** inhibition. In **(A–C)**, an average response histogram to 7, 6, and 8 efferent shock trains (each 333 shocks/s for 5 s, green column at *t* = 0–5 s) were constructed for the three different afferents, respectively, from three different animals. Dashed horizontal lines represent the prestimulus baseline afferent firing rate. Binning in all panels is 500 ms.

Mouse vestibular afferents exhibit multiple response components to electrical stimulation of the efferent vestibular system (EVS). The most common response, observed in nearly all afferent recordings, is an efferent-mediated slow excitation (Figure 1A) that takes seconds to develop and persists for tens of seconds after termination of the stimulus. The kinetics and pharmacology of this response are consistent with an efferent-mediated activation of mAChRs (Holt et al., 2017; Ramakrishna et al., 2020; Schneider et al., 2021). The second most common response, seen in approximately one-third of our afferent recordings, is an efferent-mediated fast excitation with peak amplitudes ranging from 5 to 75 spikes/s. Efferent-mediated fast excitation peaks within the first 500-ms of the stimulus and quickly returns to baseline upon stimulus termination (Figure 1B). While it can occur in isolation, efferent-mediated fast excitation, as the example shows, typically develops in tandem with efferent-mediated slow excitation. The kinetics and pharmacology of efferent-mediated fast excitation are in line with the activation of α4β2*nAChR (Holt et al., 2015; Schneider et al., 2021). Lastly, in less than 1% of our afferent recordings, an efferent-mediated fast inhibition is observed (Figure 1C). Similar to efferent-mediated fast excitation, the kinetics of efferent-mediated inhibition closely follow the onset and termination of the efferent stimulus. Given its infrequent observations in mouse, the pharmacology of efferent-mediated inhibition of vestibular afferents has not been well characterized. However, its similarity with efferent-mediated afferent inhibition in other vestibular preparations suggests it is mediated by the activation of α9α10nAChRs and SK potassium channels in type II hair cells (Sugai et al., 1992; Holt et al., 2001, 2015; Parks et al., 2017). Patch clamp recordings confirm that both components are present in mouse type II vestibular hair cells (Poppi et al., 2018, 2020; Yu et al., 2020), but it has not been pharmacologically confirmed in afferent recordings during efferent stimulation. The prevalence of α9α10nAChRs in mouse type II hair cells suggests that efferent-mediated inhibition should be more common, but it may be obscured in many of our afferent recordings after summating with the ongoing efferent-mediated fast and slow excitation. During the prestimulus time domain where the three efferent-mediated responses can overlap, selective cholinergic blockers would be helpful in isolating each EVS-activated mechanism.

### Drug Access and Charged mAChR Antagonists

Understanding what cholinergic drugs can actually reach the intact inner ear and their resulting dose-response relationships will be key in isolating specific efferent synaptic mechanisms as well as probing the potential functional roles that each of these mechanisms play in vestibular-related behaviors. Previously-characterized cholinergic drugs, when administered systemically (IP), are known to enter the CNS and also block EVS-mediated responses in the ear (Schneider et al., 2021). In the current study, in what was intended to be a negative control, we sought to determine whether the inverse would be true. Would cholinergic drugs, having little to no CNS entry, fail to enter the inner ear to block EVS-mediated afferent responses?

#### Intraperitoneal Administration of Charged mAChR Antagonists

**FIGURE 2:**
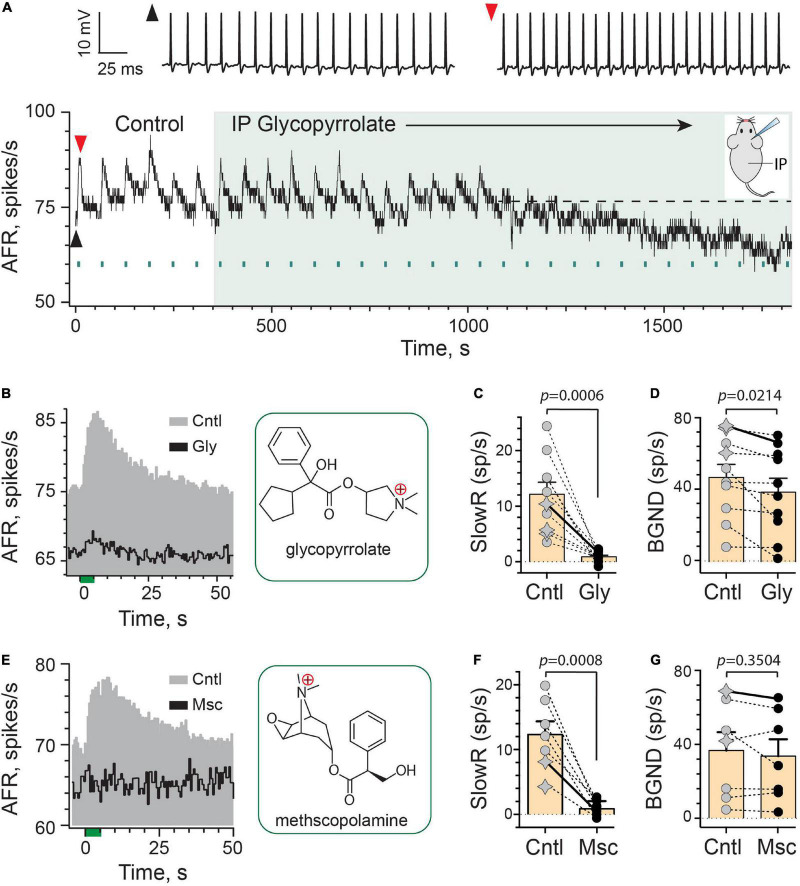
Efferent-mediated slow excitation of vestibular afferents is antagonized by peripheral mAChR antagonists. **(A)** Continuous response histogram from a regular afferent shows changes in afferent firing rate (AFR) during midline efferent stimulation (green bars, 333/s for 5 s every 60 s) before and after administering glycopyrrolate (IP, 2 mg/kg) at *t* = 360 s (green box). Raw spike data from baseline (black arrowhead) and peak efferent-mediated slow excitation (red arrowhead) are shown above the histogram. Inset: mouse diagram – afferent recording from right ear during IP drug delivery. **(B)** Corresponding average response histograms from the same afferent in **(A)** were generated separately for 6 efferent shock trains during control conditions (Cntl) and 10 trials starting at *t* = 1,150 s during IP glycopyrrolate (Gly). Chemical structure for glycopyrrolate is shown in green box. **(C,D)** Mean peak slow excitation (SlowR) and background discharge rates (BGND) are plotted for 10 afferents from 10 animals before (Cntl, gray) and after IP glycopyrrolate (Gly, black). Star symbols and filled circles in control column indicate regular and irregular afferents, respectively. Orange bars with error bars reflect the population mean and SEM. Solid line shows values from histograms in **(B)**. Indicated *p*-values from paired *t*-test. **(E)** Average response histograms showing the effects of midline efferent stimulation in an irregular afferent before (Cntl, gray) and after IP administration of 2 mg/kg methscopolamine (Msc, black). Chemical structure for methscopolamine is shown in green box. **(F,G)** Mean peak slow excitation (SlowR) and background discharge rates (BGND) are plotted for seven afferents from seven animals before (Cntl, gray) and after IP methscopolamine (Msc, black). Orange bars with error bars reflect the population mean and SEM. Solid line shows values from histograms in **(E)**. Indicated *p*-values from paired *t*-test. Binning in **(A,B,E)** is 500 ms.

To test this idea, we first characterized extracellular spike responses of mouse vestibular afferents during EVS stimulation before and after the systemic administration of the peripherally-acting mAChR antagonist glycopyrrolate (Figures 2A–D). Glycopyrrolate has a singly-charged, quaternary ammonium head (Figure 2B), which significantly limits its ability to cross the BBB (Proakis and Harris, 1978; Kaila et al., 1990; Chabicovsky et al., 2019). A continuous rate histogram from a regularly-discharging vestibular afferent is shown in Figure 2A. Repeated electrical stimulation of vestibular efferents in the brainstem (333 shocks/s for 5 s, multiple green bars) routinely elicited an excitation characterized as slow given its time to peak and return to baseline (Figures 2A,B). Note the shortening of interspike intervals in raw spike traces. At the 350-s mark, glycopyrrolate (2 mg/kg) was administered via an intraperitoneal (IP) injection. Following IP glycopyrrolate (green-shaded region), the amplitude of efferent-mediated slow excitation remained relatively unchanged for another 6–7 min, but then began to exhibit some variability in subsequent trials that fell short of matching control responses. After just over 12 min post IP glycopyrrolate, efferent stimuli elicited little to no slow excitation suggesting that glycopyrrolate does in fact reach mAChRs in the inner ear. This is nearly double the 6–8 min previously observed for blockade of efferent-mediated responses by atropine, scopolamine, and DHβE (Schneider et al., 2021). Baseline discharge rates also began to fall about the same time. The average response histograms for this unit show the differences in discharge rates during baseline and peak slow excitation (Figure 2B), where a mean slow excitation of ∼10 spikes/s was almost completely blocked and the baseline fell by about 10 spikes/s. In ten animals, IP glycopyrrolate was tested in 10 afferents (3 regular, 7 irregular) where it significantly blocked 93% of the mean efferent-mediated slow excitation [12.1 ± 2.1 vs. 0.9 ± 0.3 spikes/s, *t*(9) = 5.138, *d* = 1.625; Figure 2C] and significantly reduced baseline discharge rates [47.0 ± 7.3 vs. 38.8 ± 7.7 spikes/s, *t*(9) = 2.780, *d* = 0.8791; Figure 2D]. Blockade of slow excitation and baseline reduction with glycopyrrolate are similar to previous observations with IP administration of the mAChR antagonists atropine and scopolamine (Schneider et al., 2021).

We were surprised that glycopyrrolate was able to make its way into the inner ear given its reported restricted access to the CNS. These observations suggested the rules governing the entry of some drugs to the ear vary from the brain, presumably-based on relative differences in blood–brain barrier (BBB) and blood-labyrinth barrier (BLB) permeability. We wondered whether such entry was applicable to other charged mAChR antagonists. To test further, we employed the use of methscopolamine, which like glycopyrrolate also has a single, positively-charged, quaternary ammonium group (Figure 2E), and does not readily cross the BBB (Domino and Corssen, 1967; Freedman et al., 1989; Callegari et al., 2011). Systemically-administered methscopolamine, at doses of 2–10 mg/kg, fails to block central mAChRs in a number of experimental preparations including rats (Westerberg and Corcoran, 1987; Roth et al., 1989; Dringenberg and Vanderwolf, 1996) and mice (Lamberty and Gower, 1991; Bymaster et al., 1998; Singer and Yee, 2012; Brulet et al., 2017). Like IP glycopyrrolate, similar effects on efferent-mediated slow excitation were also seen with IP methscopolamine (Figure 2E). In seven afferents from seven animals (2 regular, 5 irregular; Figure 2F), IP methscopolamine significantly blocked nearly 92% of the mean efferent-mediated slow excitation [12.3 ± 2.0 vs. 0.9 ± 0.4 spikes/s, *t*(6) = 6.194, *d* = 2.529], but unlike glycopyrrolate, there was no significant difference between mean afferent background discharge rates before and after methscopolamine [36.9 ± 9.9 vs. 33.8 ± 9.2 spikes/s, *t*(6) = 1.012; Figure 2G]. Blockade of efferent-mediated slow excitation by glycopyrrolate and methscopolamine indicated that some cholinergic antagonists with poor CNS penetration can enter the ear. That methscopolamine does not consistently decrease baseline activity suggests that blockade of mAChRs underlying efferent-mediated slow excitation are not always tied to decreases in baseline activity. These observations also suggest that methscopolamine may have limited access to or interactions with the mechanism(s) underlying changes in baseline discharge seen with IP glycopyrrolate. This, in turn could be related to differences in the chemical structures of these two mAChR antagonists.

#### Intrabullar Administration of Charged mAChR Antagonists

**FIGURE 3:**
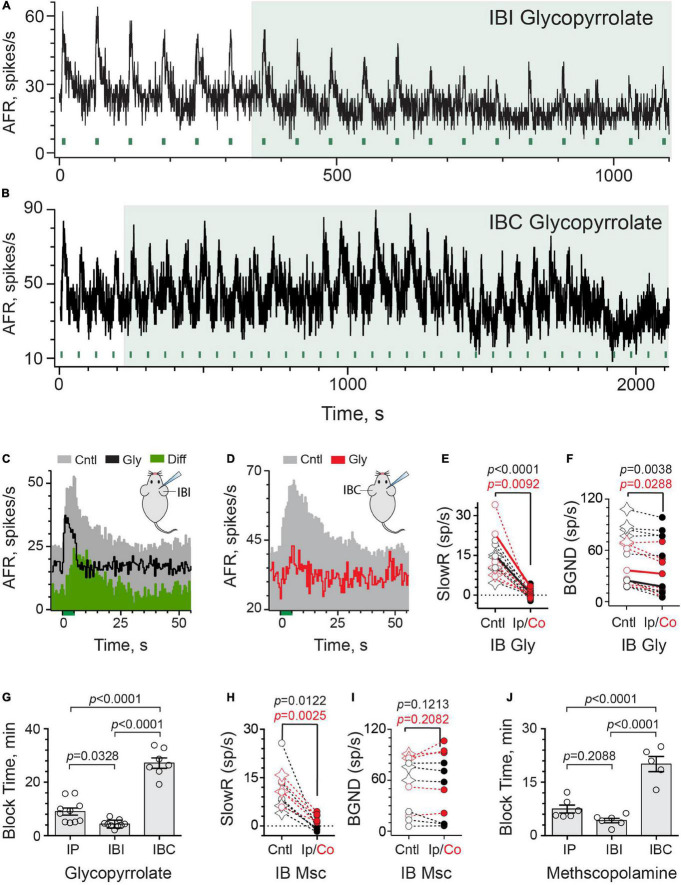
Intrabulla application of glycopyrrolate and methscopolamine also blocks efferent-mediated slow excitation in mouse vestibular afferents. **(A)** Continuous response histogram from an irregular afferent shows changes in afferent firing rate (AFR) during midline efferent stimulation (green bars, 333/s for 5 s every 60 s) before and after the ipsilateral intrabulla delivery of glycopyrrolate (30 μl at 0.5 mM), starting at *t* = 360 s (green box). **(B)** Continuous response histogram from an irregular afferent shows changes in afferent firing rate (AFR) during midline efferent stimulation (green bars, 333/s for 5 s every 60 s) before and after the contralateral intrabulla delivery of glycopyrrolate (30 μl at 0.5 mM), starting at *t* = 220 s (green box). **(C)** Corresponding average response histograms from the same afferent in **(A)** were generated separately for 6 efferent shock trains delivered before and after block by ipsilateral IB glycopyrrolate (Gly). The afferent unit displayed both fast and slow excitation and IP glycopyrrolate blocked the slow with no change on the fast. The green difference histogram, had by subtracting the Gly trace from the Cntl trace, reveals the glycopyrrolate-sensitive slow excitation. **(D)** Corresponding average response histograms from the same afferent in **(B)** were generated separately for 23 and 5 efferent shock trains, delivered before and after block by contralateral IB glycopyrrolate (Gly), respectively. **(E,F)** Values of mean peak slow excitation and background rates, respectively, during control (Cntl) and ipsilateral IB (black, Ip) or contralateral IB glycopyrrolate (red, Co). Star symbols and filled circles in control column indicate regular and irregular afferents, respectively. Solid black and red line show values from histograms in **(C,D)**, respectively. Indicated *p*-values from paired *t*-test. **(G)** Times to maximum block for IP, IBI, and IBC glycopyrrolate are compared. Indicated *p*-values from one-way ANOVA. **(H,I)** Values of mean peak slow excitation and background rates, respectively, during control (cntl) and ipsilateral IB (black, Ip) or contralateral IB methscopolamine (red, Co). Star symbols and filled circles in control column indicate regular and irregular afferents, respectively. Indicated *p*-values from paired *t*-test. **(J)** Times to maximum block for IP, IBI, and IBC methscopolamine are compared. Indicated *p*-values from one-way ANOVA. Binning in **(A–D)** is 500 ms.

We next asked whether glycopyrrolate and methscopolamine might also block efferent-mediated slow excitation if they were instead delivered to the middle ear using an intrabullar (IB) route. Many drugs, when placed in the middle ear, move across the round window membrane (RWM) into the perilymphatic compartment where they can directly interact with inner ear tissues (McCall et al., 2010; Salt and Hirose, 2018; Salt and Plontke, 2018; Patel et al., 2019). This approach, previously characterized for atropine and scopolamine (Schneider et al., 2021), delivers drugs to the inner ear faster than IP administration and may offer an opportunity to avoid/delay systemic off-target effects potentially including drug-mediated decreases in baseline discharge. Secondly, the IB route bypasses the BLB and previous evidence has revealed that the round window behaves as a semipermeable membrane where positively-charged molecules can cross quite easily (Goycoolea and Lundman, 1997; Goycoolea, 2001; Liu et al., 2013). To better understand how IB glycopyrrolate may enter the mouse ear and delineate local versus systemic access, we characterized both the effects of ipsilateral (IBI) and contralateral (IBC) delivery of glycopyrrolate, relative to the right side from which afferent recordings were made (Figures 3A,B). The continuous rate histogram in Figure 3A demonstrates the effects of IBI glycopyrrolate (30 μl @ 0.5 mM, large green box) on an irregularly-discharging afferent’s response to repeated efferent shock trains (333 shocks/s for 5 s, green bars). In this particular example, efferent stimulation elicited both a fast and slow excitation. The efferent-mediated fast excitation can be identified as the immediate jump in firing rate at the beginning of the stimulus (Figure 3C, green bar at *t* = 0). We specifically chose this record to demonstrate that glycopyrrolate, while it completely antagonized efferent-mediated slow excitation, had little effect on efferent-mediated fast excitation. Similar pharmacological observations were made with glycopyrrolate in two other units showing both efferent-mediated fast and slow excitation. In the unit shown, the first observation that glycopyrrolate is affecting the size and shape of the efferent-mediated slow excitation is 4–5 min after drug delivery. That IBI glycopyrrolate was blocking efferent-mediated slow excitation in this example is revealed in the difference histogram (Diff), generated by subtracting the Gly histogram from the Cntl histogram. As was the case with IP glycopyrrolate, IBI glycopyrrolate also reduced background discharge.

Surprisingly, contralateral IB glycopyrrolate (IBC) also produced similar blockade of efferent-mediated slow excitation and reduction of background discharge rates (Figures 3B,D), but the time to these effects was much more protracted than with either IBI or IP administration. Note, in this unit, that it takes nearly 20 min before IBC glycopyrrolate begins to impact the amplitude of efferent-mediated slow excitation and reduce background discharge. This presumably reflects the time it takes for glycopyrrolate to move from the contralateral to the ipsilateral ear. These observations also indicate that the changes in response amplitude and baseline firing are related to the arrival of glycopyrrolate and not some time-dependent decline in afferent viability. In 16 animals, the effects of IB glycopyrrolate were characterized in 16 afferents (6 regular, 10 irregular; Figure 3E), where it significantly blocked almost 98% of efferent-slow excitation using either the IBI [13.9 ± 1.6 vs. 0.3 ± 0.6 spikes/s, *t*(8) = 8.081, *d* = 2.694] or IBC route [15.2 ± 3.8 vs. 0.4 ± 0.7 spikes/s, *t*(6) = 3.782, *d* = 1.429]. Background discharge rates (Figure 3F) were also significantly reduced in both IBI [55.1 ± 11.6 vs. 48.2 ± 12.4 spikes/s, *t*(8) = 4.031, *d* = 1.344] and IBC animals [43.2 ± 8.7 vs. 33.9 ± 8.9 spikes/s, *t*(6) = 2.861, *d* = 1.081].

#### Comparison of Block Times for Intraperitoneal and Intrabullar Routes

Our motivation to use IBC glycopyrrolate was sparked by questions about how IBI glycopyrrolate arrives in the ipsilateral perilymphatic space. Conventional thinking would suggest that, upon delivery, it moves across the round and/or oval windows into the perilymphatic fluid and then diffuses to block mAChRs in the vestibular neuroepithelium (Salt and Hirose, 2018; Salt et al., 2018b; Patel et al., 2019). Alternatively, its entry into local vascular components might also rapidly deliver it to the ear in a manner similar to IP administration, but presumably faster given its proximity. Comparable entry into the local vasculature of the contralateral ear might be expected to arrive at the ipsilateral ear after some short delay needed for delivering the drug via the bloodstream. That delay might be longer if the drug must first enter the contralateral perilymph before re-entering the systemic circulation. We reasoned that these two access scenarios (i.e., round window versus local vasculature entry) could be distinguished by determining if differences existed in arrival times to the ipsilateral ear between IBI and IBC administration. Block times for IP, IBI, and IBC glycopyrrolate were revealing in this regard (Figure 3G). First, consistent with previous observations with atropine and scopolamine (Schneider et al., 2021), glycopyrrolate is significantly faster when given IBI than when given via the IP route (4.3 ± 0.5 vs. 9.0 ± 1.3 min) and both IP and IBI were significantly faster than IBC (27.2 ± 1.9 min) as determined by one-way ANOVA [*F*(2,23) = 77.29, *p* < 0.0001]. The threefold difference in block times between IBI and IBC administration is consistent with glycopyrrolate gaining direct access to the perilymphatic compartment through the round/oval windows, but the fact that IBC glycopyrrolate also reached the ipsilateral ear suggests it does eventually gain vascular access. The difference in timing may also be dependent on glycopyrrolate’s effective concentration in the ipsilateral ear as a function of where it was administered. Systemic redistribution following IBC administration should result in lower glycopyrrolate concentrations reaching the ipsilateral side, which has been seen with fluorescein (Salt et al., 2018a).

Similar experiments for IB methscopolamine were performed and characterized in 11 afferents (4 regular and 7 irregular) from 11 animals (Figures 3H,I). IB methscopolamine significantly blocked 99% of efferent-mediated slow excitation using the IBI route [11.1 ± 3.2 vs. −0.9 ± 0.4 spikes/s, *t*(5) = 3.831, *d* = 1.564] and 77% using the IBC route [11.4 ± 1.7 vs. 2.6 ± 0.6 spikes/s, *t*(4) = 6.729, *d* = 3.009]. The effects of methscopolamine on background discharge rates, however, were neither as pronounced nor consistent as glycopyrrolate. Background rates were not significantly different before and after IBI [43.0 ± 13.4 vs. 38.8 ± 14.2 spikes/s, *t*(5) = 1.864] or IBC methscopolamine (68.6 ± 13.3 vs. 74.6 ± 15.5 spikes/s, *t*(4) = 1.499) (Figure 3I). Block times for IP, IBI, and IBC methscopolamine are also presented (Figure 3J). While block times for IP (7.5 ± 1.1 min) and IBI methscopolamine (4.2 ± 0.6 min) were not significantly different, both IP and IBI routes were shorter than IBC (19.6 ± 1.8 min) as determined using a one-way ANOVA [*F*(2,15) = 39.44, *P* < 0.0001]. Again, consistent with a direct entry into the inner ear, ipsilateral IB methscopolamine was faster than contralateral IB administration.

Despite their presumed limited access to the CNS, it is clear from the data above that methscopolamine, regardless of the route of administration or time to blockade, does not significantly impact background discharge in the same way that glycopyrrolate does. With all three administration routes (i.e., IP vs. IBI vs. IBC), glycopyrrolate significantly decreased background discharge while methscopolamine did not. With methscopolamine, the effects on background discharge were quite variable. Since mammalian efferent-mediated afferent responses are typically larger in irregularly-discharging afferents than in regularly-discharging afferents (Goldberg and Fernández, 1980; Marlinski et al., 2004; Schneider et al., 2021), it might be argued that the effects of mAChR antagonists on background discharge, if related to activation of the same mAChRs, might also be larger in irregular afferents and that an overrepresentation of regularly-discharging afferents in a sampling population might mask detection of significant mean differences between control and post-drug background discharge rates. As seen in Figures 2D,G, 3F,I, about one-third of units (15 of 44) are regularly-discharging suggesting that they are not particularly overrepresented. However, to identify if significant differences in background discharge rates exist between regular and irregular afferents following glycopyrrolate or methscopolamine, we pooled the data from all three administration routes for each drug and then separated them into regular (CV* < 0.1) and irregular (CV* > 0.1) groups. Pooling was justified given their significant effect (i.e., glycopyrrolate) or lack thereof (i.e., methscopolamine) on mean background discharge rates among all three administration routes. Background rates before and after glycopyrrolate were significantly different in both regular [78.0 ± 4.6 vs. 72.5 ± 4.6 spikes/s, paired *t*-test, *p* = 0.0290, *t*(8) = 2.655, *d* = 0.9386] and irregular afferents [33.3 ± 4.2 vs. 23.9 ± 4.3 spikes/s, paired *t*-test, *p* = 0.0002, *t*(17) = 4.731, *d* = 1.183], while background rates before and after methscopolamine were not significantly different in either regular [71.1 ± 7.4 vs. 73.8 ± 9.1 spikes/s, paired *t*-test, *p* = 0.5115, *t*(7) = 0.7063] or irregular afferents [39.2 ± 8.2 vs. 37.0 ± 8.7 spikes/s, paired *t*-test, *p* = 0.3619, *t*(11) = 0.9349]. These observations suggest that the different effects of glycopyrrolate and methscopolamine on an afferent’s background discharge does not seem to be related to differences in discharge regularity.

#### Intracanal Administration of Charged mAChR Antagonists

**FIGURE 4:**
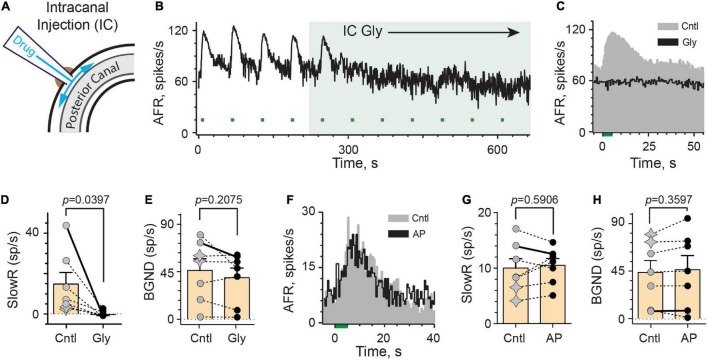
Intracanal application of glycopyrrolate rapidly blocks efferent-mediated slow excitation in mouse vestibular afferents. **(A)** Our intracanal perilymphatic injection (IC) is made possible by inserting and sealing a small plastic tube within the bony wall of the posterior canal. When connected to a Hamilton syringe, 1–2 μl volumes can be slowly delivered to the perilymph within the posterior canal to then diffuse to the vestibule and cochlea. **(B)** Continuous response histogram from an irregular afferent shows changes in afferent firing rate (AFR) during midline efferent stimulation (green bars, 333/s for 5 s every 60 s) before and after perilymphatic injection of glycopyrrolate (1 μl at 0.5 mM) through the bony posterior canal wall. Glycopyrrolate was slow injected over 30 s starting at *t* = 220 s (green box). **(C)** Corresponding average response histograms from the same afferent in **(B)** were generated separately for the first four and last five efferent shock trains, delivered before (Cntl) and after IC glycopyrrolate (Gly), respectively. **(D,E)** Values of mean peak slow excitation (SlowR) and background discharge rates (BGND), respectively, during control (Cntl) and IC glycopyrrolate (Gly). Star symbols and filled circles in control column indicate regular and irregular afferents, respectively. Orange bars with error bars reflect the population mean and SEM. Solid line shows values from histograms in **(C)**. Indicated *p*-value from paired *t*-test. **(F)** Average response histograms for another afferent were generated for efferent shock trains, delivered before (Cntl) and after the IC injection of artificial perilymph (AP). Star symbols and filled circles in control column indicate regular and irregular afferents, respectively. **(G,H)** Values of mean peak slow excitation (SlowR) and background discharge rates (BGND), respectively, during control (Cntl) and IC artificial perilymph (AP). Orange bars with error bars reflect the population mean and SEM. Solid line shows values from histograms in **(F)**. Indicated *p*-value from paired *t*-test. Binning in **(B,C,F)** is 500 ms.

As governed by its ability to block efferent-mediated slow excitation of vestibular afferents, glycopyrrolate and methscopolamine eventually reach the perilymphatic compartment following intraperitoneal injection or delivery into either middle ear. Glycopyrrolate also significantly reduces background discharge via IP or IB routes suggesting that it might alter afferent excitability through some peripheral mechanism. In order to confirm that these effects are specifically related to the delivery of glycopyrrolate to the inner ear, we administered the drug directly into the perilymph using an intracanal (IC) injection. After making a small hole in the wall of the bony posterior canal, the tip of a polypropylene microcannula, connected to a Hamilton syringe filled with glycopyrrolate in artificial perilymph (AP), was inserted and sealed in place using cyanoacrylate glue (Figure 4A). In the continuous rate histogram shown (Figure 4B), efferent shock trains (333 shocks/s for 5 s, multiple green bars) repeatedly elicited a large slow excitation in an irregularly-discharging vestibular afferent. After the fourth efferent stimulus, 1 μl of glycopyrrolate (0.2 mg/ml in AP; 0.5 mM) was injected over 30 s near the 220-s mark (green-shaded box). The afferent response to the first efferent stimulus following IC glycopyrrolate was unremarkable and appears comparable to those observed during control conditions. However, by the second post-drug efferent shock train, the afferent failed to respond to efferent stimulation and efferent-mediated slow excitation remains blocked for subsequent efferent stimuli. The average response histograms demonstrate that a peak slow excitation of more than 40 spikes/s is completely blocked following the IC injection (Figure 4C). In seven animals, the effects of IC glycopyrrolate were characterized in seven afferents (2 regular and 5 irregular) where it significantly blocked 98% of efferent-mediated slow excitation [14.7 ± 5.7 vs. −0.3 ± 0.5 spikes/s, *t*(6) = 2.617, *d* = 0.9891] (Figure 4D). The mean time to block for the IC route was 2.4 ± 0.6 min which was significantly shorter than the IBI route (*p* = 0.0240, Unpaired *t*-test). While a baseline reduction was associated with IC glycopyrrolate in the example shown in Figure 4B, this effect was not consistent and there was not a significant reduction in background activity before and after glycopyrrolate across the seven units [46.0 ± 10.8 vs. 39.1 ± 9.1 spikes/s; *t*(6) = 1.413; Figure 4E]. Importantly, as a control measure, IC injection of artificial perilymph (AP) had little effect on efferent-mediated slow excitation (Figure 4F). In seven units (2 regular and 5 irregular) from four animals, IC AP had no significant effect on efferent-mediated slow excitation [9.9 ± 1.7 vs. 10.5 ± 1.2 spikes/s, *t*(6) = 0.5681; Figure 4G] or background discharge [43.4 ± 11.2 vs. 46.0 ± 13.3 spikes/s, *t*(6) = 0.9916; Figure 4H]. Collectively, these data indicate that blockade of efferent-mediated slow excitation by glycopyrrolate, regardless of administration route, is attributed to blockade of mAChRs in the inner ear.

### Assessing the Entry of Quaternary Alpha9-nAChR Antagonists Into the Inner Ear

**FIGURE 5:**
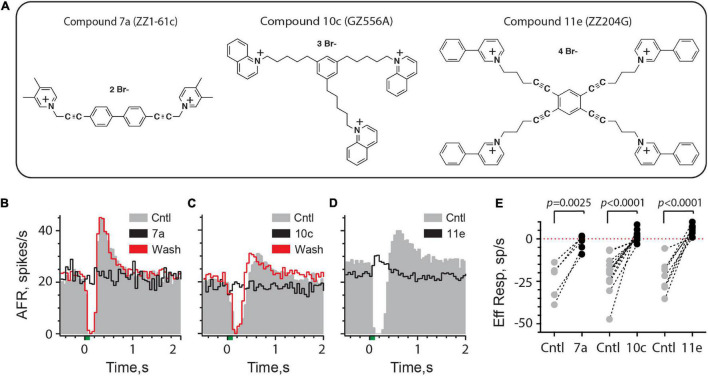
Efferent-mediated inhibition of turtle vestibular afferents is antagonized by charged α9α10nAChR blockers. **(A)** Chemical structures of bis, tris, and tetrakis quaternary ammonium compounds 7a, 10c, and 11e are shown (see also Zheng et al., 2011). **(B–D)** Average response histograms showing the effects of efferent stimulation in turtle bouton afferents before (Cntl, gray) and during the application of Compound 7a, 10c, or 11e (Black trace), respectively. Efferent-mediated inhibition was recovered after washout (Wash, red trace) with Compounds 7a and 10c. Efferent shock trains (20 shocks at 200/s, green bar at *t* = 0) were repeated every 3 s and all histograms were based on at least 20 shock train presentations. **(E)** Mean afferent responses to efferent stimulation (Eff Resp) are plotted for multiple afferents before (Cntl, gray-filled circles) and after the application of Compound 7a, 10c, or 11e (black-filled circles). Negative values indicate inhibition while positive values reveal efferent-mediated excitatory effects. Indicated *p*-values from comparisons made using a paired *t*-test. Binning in **(B–D)** is 50 ms.

These observations suggest we may be able to identify a number of additional charged drugs with the ability to block a host of synaptic mechanisms in the inner ear without targeting similar components in the brain. We also wanted to explore if charged nAChR antagonists could be used to investigate efferent-mediated inhibition or efferent-mediated fast excitation of vestibular afferents. To this end, Zheng et al. (2007, 2011) and López-Hernández et al. (2009) have developed a series of novel *bis*-, *tris*-, and *tetrakis*-azaaromatic quaternary ammonium analogs that function as potent nAChR antagonists. On basis of potency and selectivity, three compounds, referred to as Cmpd7a, 10c, and 11e (Figure 5A), were shown to block α9α10nAChR-mediated responses in *Xenopus* oocytes (Zheng et al., 2011). However, their effectiveness at blocking α9α10nAChRs in the inner ear has not been characterized. In order to better understand how these analogs could be used in this regard, we asked several broad questions: (1) Are these compounds, in fact, potent inhibitors of efferent-mediated processes in the inner ear attributed to activation of α9α10nAChRs?; (2) How quickly do they block? and (3) Because Cmpd7a, 10c, and 11e possess two, three, or four quaternary ammonium heads, respectively, could they block α9α10nAChR-mediated responses in the mammalian inner ear if administered systemically, in a manner similar to blockade of mAChRs with glycopyrrolate and methscopolamine?

#### Charged α9α10nAChR Antagonists Block Efferent-Mediated Inhibition of Turtle Vestibular Afferents

To answer the first two questions, we first characterized the effects of Cmpd7a, 10c and 11e on efferent-mediated inhibition of vestibular afferents innervating the turtle posterior crista. This preparation is advantageous in that it allows direct drug application to the crista neuroepithelium while electrically stimulating vestibular efferent neurons, and that the pharmacology of the underlying efferent mechanisms are well understood (Holt et al., 2006, 2015, 2017; Parks et al., 2017). With direct drug access, we can also approximate minimal blocking concentrations and the length of time needed to achieve complete blockade, which are useful benchmarks for probing similar mechanisms in mice.

Efferent-mediated inhibition in turtle may be purely inhibitory or followed by a post-inhibitory excitation (PIE), but both responses are blocked by α9α10nAChR antagonists including strychnine, tropisetron, α-bungarotoxin, and α-conotoxin RgIa (Holt et al., 2006, 2015). Furthermore, PIE is mostly dependent on the preceding inhibition and will also be blocked by α9α10nAChR antagonists. Consistent with its effect on α9α10nAChRs in Xenopus oocytes (Zheng et al., 2011), superfusion of Cmpd7a, at a concentration of 100 nM, completely blocked both efferent-mediated inhibition and the subsequent PIE in a turtle afferent (Figure 5B). The blockade was reversible as evidenced by return of the efferent-mediated inhibition and PIE during the washout period. The effect of Cmpd7a (0.1–1 μM) was evaluated in five afferents from four animals (Figure 5E), where it significantly blocked 89% of efferent-mediated inhibition [−24.7 ± 4.7 vs. −2.8 ± 2.0 spikes/s, *t*(4) = 6.737, *d* = 3.013]. Similar observations in 12 afferents from 7 animals were made for Cmpd10c (Figures 5C,E) whose superfusion at concentrations of 0.1–1 μM significantly blocked 108% of efferent-mediated inhibition [−20.8 ± 3.2 vs. 1.6 ± 0.9 spikes/s, *t*(11) = 5.922, *d* = 1.710]. The greater than 100% blockade is a reflection of efferent-mediated fast excitation which is often unmasked after applying α9α10nAChR antagonists (Holt et al., 2015). This excitation can be identified in several units where the post-blockade values in Cmpd10c are above the zero-line indicating that the direction of the response has reversed (Figure 5E). Finally, consistent with previous characterization in Xenopus oocytes (Zheng et al., 2011), the most potent of the three analogs was Cmpd11e which completely blocked efferent-mediated inhibition in our preparation down to concentrations as low as 10 nM. In the example shown in Figure 5D, 100 nM Cmpd11e completely antagonized the inhibitory response and unmasked an efferent-mediated excitatory response. The effects of Cmpd11e (0.01–2 μM) were tested in nine afferents from 5 animals (Figure 5E), where it significantly blocked 122% of the efferent-mediated inhibition [−22.5 ± 2.9 vs. 5.0 ± 1.0 spikes/s, *t*(8) = 7.97, *d* = 2.657]. Again, the unmasking of efferent-mediated excitation is reflected in the positive post-blockade values for Cmpd11e. Mean block times for 7a (11.4 ± 2.7 min), 10c (11.2 ± 1.9 min), and 11e (12.2 ± 3.1 min) were not significantly different [Kruskal–Wallis: *H*(2) = 0.1054, *P* = 0.9487). Collectively, these data demonstrate that these charged compounds are potent inhibitors of α9α10nAChRs present on vestibular type II hair cells in the turtle inner ear.

#### Quaternary α9α10nAChR Antagonists in the Mouse Inner Ear

We next sought to determine if Cmpd7a, 10c, and 11e could block α9α10nAChR-mediated responses in the mammalian inner ear if administered systemically. Our data with glycopyrrolate and methscopolamine indicated that the systemic administration of some charged compounds can make it to the inner ear. Although α9α10nAChRs are expressed in mammalian vestibular endorgans and their activation gives rise to hyperpolarization of type II hair cells (Poppi et al., 2018, 2020; Yu et al., 2020), direct observations of efferent-mediated inhibition of mouse vestibular afferents are infrequent (Goldberg and Fernández, 1980; Schneider et al., 2021; also see Figure 1A). One likely explanation is that the inhibitory component of efferent-mediated afferent responses is obscured by efferent-mediated fast excitation (Holt et al., 2015). As such, it would be experimentally challenging to identify if these quaternary α9α10nAChR antagonists, when given systemically, were in fact directly blocking α9α10nAChRs in the peripheral vestibular system.

**FIGURE 6:**
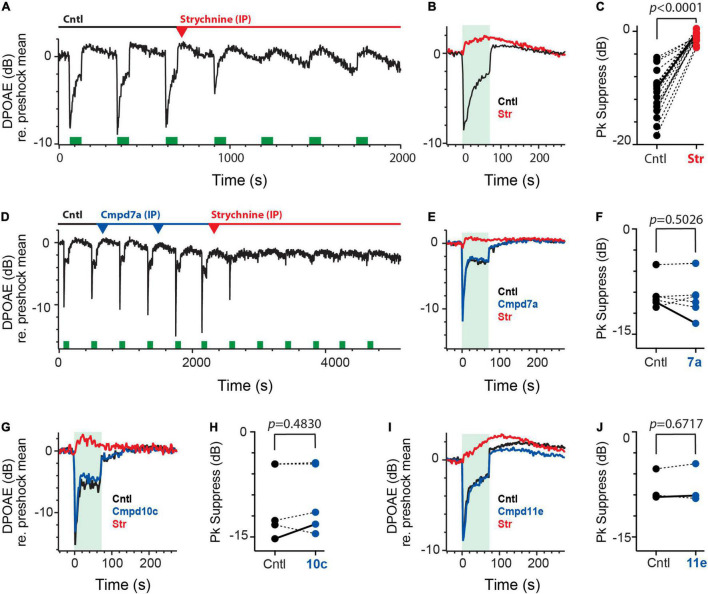
Intraperitoneal administration of charged α9α10nAChR blockers fail to block efferent-mediated suppression of mice DPOAEs. **(A)** Continuous recording of mouse DPOAEs during midline stimulation of MOC efferent neurons (green bars, 200 shock/s for 70 s), before (Cntl) and after IP administration of strychnine at *t* = 700 s. (red arrowhead, 6 mg/kg). **(B)** Mean responses, from **(A)**, showing the effect of midline efferent stimulation (70-s duration @ 200 shocks/s, green box) on DPOAE amplitude before (Cntl, black trace) and after IP administration of strychnine (Str, red). **(C)** Peak values of efferent-mediated DPOAE suppression (Pk Suppress) are plotted for multiple animals before (Cntl, black) and after the application of strychnine (Str, red). Solid line shows values from mean traces in **(B)**. **(D)** Continuous recording of mouse DPOAEs during midline stimulation of MOC efferent neurons (green bars, 200 shock/s for 70 s), before (Cntl) and after two IP doses of Cmpd7a (2.5 mg/kg each, blue arrowheads) and subsequent IP strychnine (6 mg/kg, red arrowhead). **(E)** Mean responses, from **(D)**, showing the effect of midline efferent stimulation (70-s duration @ 200 shocks/s, green box) on DPOAE amplitude before (Cntl, black trace) and after IP delivery of Cmpd7a (blue trace) and then strychnine (red trace). **(F,H,J)** Peak values of efferent-mediated DPOAE suppression (Pk Suppress) are plotted for multiple animals before (Cntl, black) and after the application of Cmpd7a, 10c, or 11e (blue). Solid line shows values from corresponding traces in **(E,G,I)**. **(G,I)** Mean responses showing the effect of midline efferent stimulation (70-s duration @ 200 shocks/s, green box) on DPOAE amplitude before (Cntl, black trace) and after IP delivery of Cmpd10c (2.5 mg/Kg, blue trace) or Cmpd11e (2.5 mg/kg, blue trace), respectively. Both are then followed by IP strychnine (6 mg/Kg, red trace). Indicated *p*-values in **(C,F,H,J)** were computed using a paired *t*-test. Binning in **(A,B,D,E,G,I)** is 2.3 s.

However, a much more reliable and robust source of α9α10nAChR activation in the inner ear can be found on the auditory side. By recording distortion product otoacoustic emissions (DPOAEs) during electrical stimulation of medial olivocochlear (MOC) efferent neurons, we can probe the activation of α9α10nAChRs on outer hair cells (OHCs). Activation of MOC neurons in mice, on average, can produce a 5–15 dB peak suppression of DPOAE levels (Maison et al., 2007; Vetter et al., 2007). That this suppression is mediated by α9α10nAChRs is supported by selective pharmacological blockade, its notable absence in α9 and α10 nAChR subunit knockout mice, and enhancement in α9 gain-of-function mutants (Vetter et al., 1999, 2007; Maison et al., 2007; Taranda et al., 2009). Using a similar approach, we wanted to characterize the effects of MOC stimulation on DPOAE levels before and after the IP administration of Cmpd7a, 10c, and 11e. Under control conditions (Figure 6A), repeated delivery of the MOC efferent shock train (200 shock/s for 70 s, green bars) resulted in a ∼8 dB peak suppression of DPOAE levels near the onset of the efferent stimulus. Over the length of the stimulus, efferent suppression of DPOAEs exhibited variable levels of decay toward baseline and the post-stimulus period was often marked by a slow enhancement in DPOAEs levels that could persist for hundreds of seconds (Figure 6B). For the 44 animals used in this study, the mean efferent-mediated suppression and enhancement of DPOAEs was −11.5 ± 0.6 dB and 1.1 ± 0.2 dB, respectively.

We wanted to confirm that the observed suppression was in fact mediated by α9α10nAChRs. At the end of the third efferent shock train, a single dose of the α9α10nAChR antagonist strychnine (6 mg/kg) was delivered by IP injection where it completely blocked the efferent-mediated suppression in just under 10 min while leaving efferent-mediated slow enhancement intact (Figures 6A,B). IP strychnine (6 mg/kg) significantly blocked 92% of the efferent suppression [−10.8 ± 0.9 vs. −0.9 ± 0.2 dB, *t*(18) = 11.57, *d* = 2.654; Figure 6C] in 19 animals without significant effects on efferent-mediated slow enhancement [0.8 ± 0.2 vs. 1.3 ± 0.4 dB, paired *t*-test, *t*(18) = 0.9489, *p* = 0.3552; data not shown]. The waveforms associated with efferent-mediated suppression and slow enhancement of DPOAEs as well as their differential sensitivity to strychnine are consistent with previous data (Maison et al., 2007).

Using the same preparation, we then asked whether the IP administration of any of the charged α9α10nAChR antagonists would also affect efferent-mediated suppression of DPOAEs. We started with Cmpd7a which exhibits a similar blocking potency as strychnine (i.e., IC50: 16 nM vs. 20 nM, respectively) in blocking α9α10nAChRs in Xenopus oocytes (Elgoyhen et al., 2001; Zheng et al., 2011). Given equipotency, we reasoned that similar IP doses of Cmpd7a should block the efferent-mediated suppression provided that the drug can actually access the inner ear in sufficient concentrations. Figure 6D shows another continuous recording of DPOAEs during efferent stimulation. Efferent shock trains (200 shocks/s for 70 s, green bars) produced a ∼10 dB peak suppression. At the 10-min mark, a single IP injection of Cmpd7a (2.5 mg/kg, blue arrowhead) failed to block the suppression to the next two efferent shock trains. At that time, a second IP injection of Cmpd7a at the same dose (2nd blue arrowhead) was administered which again failed to block the efferent-mediated suppression. Ironically, the amplitude of the efferent-mediated suppression was larger, in this example, after the second dose of Cmpd7a. As a positive control, IP strychnine (6 mg/kg, red arrowhead) was administered and blocked over half the suppression by the next shock train. Mean responses are shown in Figure 6E. Blockade by strychnine was complete in just over 10 min post-injection, consistent with the time course in Figure 6A. This suggests that the effects of strychnine blockade were not accelerated by the pretreatment with Cmpd7a, which would likely be observed if Cmpd7a had any inner ear access. Given the differences in charge, molecular weight, and structure, one might posit that the fraction of Cmpd7a that enters the inner ear after IP administration is lower than that seen with strychnine. However, doses as large as 38 mg/kg with an average exposure time near 30 min failed to block the efferent-mediated suppression. In four animals, a total of six IP injections of Cmpd7a (1–38 mg/kg) had no significant effect on efferent-mediated suppression [−9.3 ± 0.9 vs. −9.8 ± 1.2 dB, *t*(5) = 0.7221; Figure 6F] or slow enhancement [0.8 ± 0.2 vs. 0.4 ± 0.1 dB, paired *t*-test, *t*(5) = 1.888, *p* = 0.1177; data not shown]. Unlike glycopyrrolate and methscopolamine, these data suggest that Cmpd7a fails to access the inner ear when administered systemically.

If the inability of Cmpd7a to find its way into the perilymphatic compartment is attributed to its two quaternary ammonium heads, then the other charged analogs will likely be excluded as well. DPOAE suppression assays were repeated for Cmpd10c (Figures 6G,H) and Cmpd11e (Figures 6I,J), whose reported IC50 values of blocking α9α10nAChRs in Xenopus oocytes (i.e., 4.2 and 0.56 nM, respectively) are nearly 5- and 40-fold more potent than strychnine or Cmpd7a (Elgoyhen et al., 2001; Zheng et al., 2011). However, IP Cmpd10c and Cmpd11e at a range of doses were without effects on efferent-mediated suppression (Figures 6G,I). The effects of five IP injections of Cmpd10c (2.5–49 mg/kg) at exposure times ranging from 13 to 40 min were evaluated in four animals where it had no significant effect on efferent-mediated suppression [−10.1 ± 2.3 vs. −9.6 ± 2.2 dB, *t*(4) = 0.7723; Figure 6H] or changes in measurements of the slow enhancement [−0.3 ± 0.2 vs. −0.4 ± 0.2 dB, paired *t*-test, *t*(4) = 0.3582, *p* = 0.7383; data not shown]. Similarly, in three animals, IP Cmpd11e (2.5–5 mg/kg) at a mean exposure time near 40 min had no significant effect on efferent suppression [−7.9 ± 1.3 vs. −7.7 ± 1.6 dB, *t*(2) = 0.4915; Figure 6J] or slow enhancement [1.9 ± 0.6 vs. 1.3 ± 0.6 dB, paired *t*-test, *t*(2) = 1.065, *p* = 0.3984; data not shown]. In both cases, complete blockade by subsequent IP strychnine (Figures 6G,I) suggest that, like Cmpd7a, Cmpd10c, and Cmpd11e fail to reach the inner ear in sufficient concentrations to block α9α10nAChRs. Post-drug block times for strychnine in all cases were similar [11.5 ± 1.2 (Cntl) vs. 11.5 ± 2.4 (7a) vs. 9.0 ± 1.1 (10c) vs. 12.3 ± 3.9 (11e)].

**FIGURE 7:**
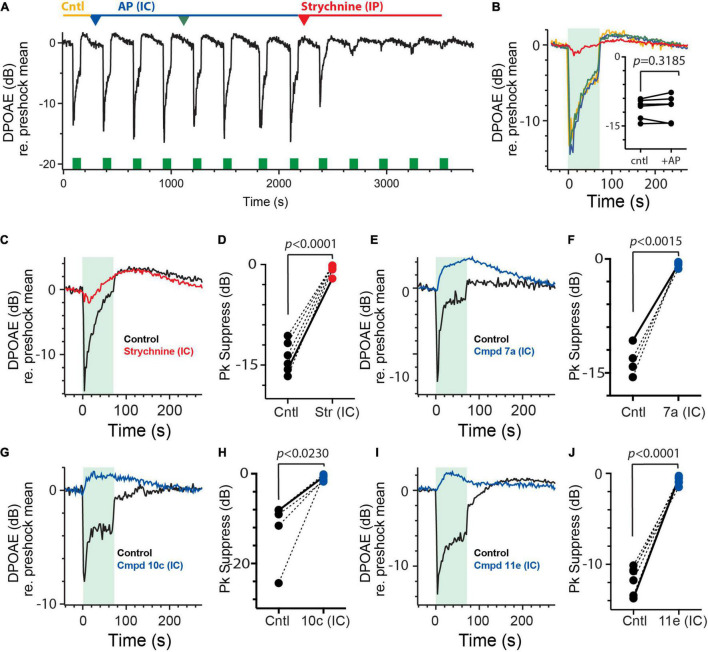
Intracanal administration of charged α9α10nAChR antagonists blocks efferent-mediated suppression of mice DPOAEs. **(A)** Continuous recording of mouse DPOAEs during midline stimulation of MOC efferent neurons (green bars, 200 shock/s for 70 s), before (Cntl) and after two IC injections of artificial perilymph (AP, 1–1.5 μl each, blue and green arrowheads) and subsequent IP strychnine (6 mg/Kg, red arrowhead). **(B)** Mean responses, from **(A)**, showing the effect of midline efferent stimulation (70-s duration @ 200 shocks/s, green box) on DPOAE amplitude before (Cntl, yellow trace) and after two IC injections of artificial perilymph (AP, blue and green traces) and subsequent strychnine (red trace). Inset: peak values of efferent-mediated DPOAE suppression are plotted for multiple animals before (Cntl) and after the IC injection of AP (+AP). Indicated *p*-values were computed using a paired *t*-test. **(C,E,G,I)** Mean responses showing the effect of midline efferent stimulation (70-s duration @ 200 shocks/s, green box) on DPOAE amplitude before (Cntl, black trace) and after IC injection of strychnine (1.5 μl @1.6 mM, red trace), Cmpd7a (1 μl @ 5 mM, blue trace), Cmpd10c (3 μl @ 2.5 mM, blue trace), or Cmpd11e (1.5 μl @ 3 mM, blue trace), respectively. **(D,F,H,J)** Peak values of efferent-mediated DPOAE suppression (Pk Suppress) are plotted for multiple animals before (Cntl, black) and after IC injection of strychnine, Cmpd7a, Cmpd10c, or Cmpd11e (red or blue trace), respectively. Solid line shows values from corresponding traces in **(C,E,G,I)**. Indicated *p*-values were computed using a paired *t*-test. Binning in **(A–C,E,G,I)** is 2.3 s.

In order to demonstrate that the failure of these compounds to block α9α10nAChR-mediated efferent suppression of DPOAEs was attributed to their inability to gain access to the inner ear, we asked if direct injection into the perilymphatic compartment would result in successful blockade. Here, we revisited the IC approach by which we can introduce small volumes of drugs into the perilymph via a small fenestra made in the bony wall of the posterior canal. While we demonstrated earlier that IC administration of artificial perilymph (AP) had no significant effect on efferent-mediated slow response of vestibular afferents (Figure 4F), we wanted to be sure that similar AP injections were without effect on efferent-mediated changes in DPOAEs. In the continuous DPOAE recording shown in Figure 7A, efferent shock trains (200 shocks/s for 70 s, green boxes) gave rise to a characteristic suppression followed by a slow enhancement. As anticipated, repeated AP injections into the posterior canal (1.5 μl each, blue/green arrowheads) failed to modify the response waveform in any consistent way, while a single IP dose of strychnine (6 mg/kg, red arrowhead) blocked the suppression. Mean responses under each condition are shown in Figure 7B. In three animals, 6 separate IC injections of AP had no significant effect on efferent-mediated DPOAE suppression [−11.2 ± 0.9 vs. 11.0 ± 1.1, *t*(5) = 0.5468; Figure 7B inset] or slow enhancement [0.6 ± 0.1 vs. 0.7 ± 0.1 dB, paired *t*-test, *t*(5) = 1.513, *p* = 0.1908; data not shown]. As a positive control that our IC drug injections do in fact reach cochlear OHCs, we also evaluated the effects of IC strychnine on efferent-mediated suppression (Figure 7C). Similar to IP administration, IC strychnine also blocks efferent-mediated suppression without effects on the slow enhancement. In six animals, IC strychnine (0.5–2.5 μl @ 1.6–3.2 mM) significantly blocked 95% of the efferent suppression [−13.8 ± 0.9 vs. −0.6 ± 0.3 dB; *t*(5) = 16.69; *d* = 6.814; Figure 7D] without any significant effects on the slow enhancement [2.7 ± 0.5 vs. 2.1 ± 0.4, paired *t*-test; *t*(5) = 1.081; *p* = 0.3290; data not shown].

The next obvious step was to administer the charged α9α10nAChR antagonists via the IC route to determine if having direct access to the perilymph results in blockade of the efferent-mediated DPOAE suppression. In contrast to IP administration, IC delivery of Cmpd7a, Cmpd 10c, and Cmpd11e blocked most of the efferent suppression of DPOAEs without any consistent effect on the slow enhancement (Figures 7E,G,I). In 4, 5, and 6 animals, Cmpd7a (1–4 μl @ 1–5 mM), Cmpd10c (0.5–3 μl @ 2.5 mM), and Cmpd11e (1–3.5 μl @ 0.3–3 mM) significantly blocked 94% [−13.4 ± 1.0 vs. −0.8 ± 0.2 dB, *t*(3) = 11.32, *d* = 6.53; Figure 7F], 93% [−12.2 ± 3.1 vs. −0.9 ± 0.3 dB, *t*(4) = 3.587, *d* = 1.604; Figure 7H], and 93% [−11.7 ± −0.6 vs. −0.8 ± 0.2 dB, *t*(5) = 14.45, *d* = 5.90; Figure 7J] of the efferent-mediated suppression, respectively. However, as with strychnine application, efferent-mediated slow enhancement was not significantly affected with either Cmpd7a [1.2 ± 0.2 vs. 1.9 ± 0.6 dB, paired *t*-test, *p* = 0.4758, *t*(3) = 0.8129], Cmpd10c [1.5 ± 0.6 vs. 0.6 ± 0.2 dB, paired *t*-test, *p* = 0.1817, *t*(4) = 1.615], or Cmpd11e [1.2 ± 0.7 vs. 1.5 ± 0.9 dB, paired *t*-test, *p* = 0.5562, *t*(5) = 0.6302] (data not shown). These data demonstrate that in order for these charged α9α10nAChR antagonists to block α9α10nAChRs in the inner ear, they must be applied directly into the perilymph, as systemic administration fails to reach the inner ear, at least with the doses and exposure times studied here.

## Discussion

We have had a long-standing interest in identifying pharmacological agents with high selectivity toward the various cholinergic receptors utilized by the peripheral EVS. Ideally, each selective drug would block the receptor in question while, depending on the dose, would have little to no effect on the others. To this end, we have previously demonstrated in the turtle posterior crista that α-bungarotoxin (αBTX), strychnine, and α-conotoxin RgIA (αCtxRgIA) potently antagonize α9α10nAChRs underlying efferent-mediated afferent inhibition, while DHβE, αCtxMII and bPiDDb potently block α4α6β2nAChRs underlying efferent-mediated fast excitation, and mAChR agents selectively targeted efferent-mediated slow excitation (Holt et al., 2006, 2015, 2017).

The turtle crista preparation is well-suited for pharmacological characterization of vestibular efferent synaptic mechanisms as these cholinergic drugs could be directly applied to the neuroepithelium without access issues. Ease of drug access has also been advantageous in characterizing efferent cholinergic mechanisms in vestibular endorgan preparations from mice (Poppi et al., 2018, 2020; Ramakrishna et al., 2020; Yu et al., 2020). However, in characterizing efferent-mediated afferent responses in the intact ear of anesthetized mice, there had to be considerations about whether some of the aforementioned drugs would make it to the perilymphatic space during systemic or middle ear administration. Our recent vestibular work as well as previous studies investigating cochlear efferents have demonstrated that many of these drugs including DHβE, atropine, scopolamine, and strychnine do, in fact, reliably access the mouse inner ear on a reasonably quick time scale (Maison et al., 2007; Schneider et al., 2021). While these drugs are tertiary amine compounds with small molecular weights (MW < 500 Da) that favor movement into the CNS as well as the ear, we questioned whether larger MW compounds like αBTX, αCtxRgIA, αCtxMII or charged drugs like bPiDDb would have similar access? We also wanted to identify if BBB permeability was a prerequisite for getting these cholinergic drugs into the ear. Selectively targeting the inner ear while avoiding confounding CNS effects would be instrumental in probing efferent synaptic mechanisms in behaving animal models, particularly for vestibular efferents where there is still debate as to what their physiological role is (Raghu et al., 2019; Cullen and Wei, 2021; Schneider et al., 2021). Identification of ear-specific drugs combined with local administration strategies could be helpful in this regard.

### Predicting Inner Ear Drug Access

A comprehensive, computational model was recently made available at the SwissADME website^1^ that consider a drug’s physiochemical properties (e.g., lipid solubility, size, polar surface, etc.) and pharmacokinetic profile to predict how readily that drug may pass through biological membranes, with a focus on the BBB and absorption in the gut (Daina and Zoete, 2016; Daina et al., 2017). Recent studies characterizing drug entry into the cochlea have made good use of this model (Salt et al., 2019; Walia et al., 2021), with the reasonable assumption that drugs demonstrating BBB permeability might be expected to also penetrate the BLB during systemic administration or the round window during middle ear application. Based on their chemical structures, the online portal predicts that DHβE, atropine, scopolamine, and strychnine will have access to the CNS and presumably the inner ear, a prediction certainly confirmed in this and previous physiological studies (Maison et al., 2007; Schneider et al., 2021).

However, the SwissADME model indicates that the singly-charged mAChR antagonists glycopyrrolate and methscopolamine should not cross the BBB, and yet in this study we demonstrated they both can enter the perilymph to block efferent-mediated slow excitation after both IP and IB administration. Similar observations apply to the ionic tracer TMPA (Inamura and Salt, 1992; Mikulec et al., 2009). Collectively, these results demonstrate that BBB permeability is not necessarily the only predictor for entry into the ear. In contrast, the charged AMPA receptor antagonist IEM1460 was found in both CSF and perilymph following IP administration, in agreement with SwissADME predictions (Walia et al., 2021). These observations suggest that additional properties simply beyond MW and polarity influence what drugs enter the CNS, ear, or both. Last but not least, we also explored whether the bis, tris, and tetrakis quaternary ammonium α9α10nAChR antagonists Cmpd7a, 10c, and 11e could be used to block efferent-mediated suppression of DPOAEs. The multiple quaternary ammonium heads suggest that they will exhibit low to no BBB permeability (Wala et al., 2012; Walbaum, 2017). The SwissADME may be less useful here in that the molecular weights and structures of Cmpd7a, 10c, and 11e exceed some of the defined parametric ranges thus rendering subsequent predictions suboptimal. That being said, these drugs were only effective in blocking α9α10nAChR-mediated cochlear responses when directly injected into the perilymphatic compartment, but not when given systemically. These observations are consistent with an inability of these drugs to cross the BBB and BLB, at least within the time frame studied here.

In a limited survey of charged compounds that access the inner ear, those that gain access (i.e., glycopyrrolate, methscopolamine, and IEM1460) are aliphatic quaternary ammonium analogs while the α9α10nAChR antagonists Cmpd7a, 10c, and 11e are aromatic quaternary ammonium analogs. There are key differences in how aliphatic and aromatic compounds interact with lipid membranes as well as a variety of transporter and efflux mechanisms that might facilitate drug permeability (Metzner et al., 2006; Geldenhuys et al., 2010; Lind et al., 2021). Whether some of these differences contribute to the entry or efflux of these cholinergic drugs in or out of the inner ear and brain remains to be determined, but some cholinergic drugs are substrates for choline transporter uptake and *P*-glycoprotein-mediated efflux which may heavily influence drug accumulation in a particular compartment (Daneman et al., 2010; Geldenhuys et al., 2010; Wakuda et al., 2019).

It will be important to quantify the concentrations of cholinergic drugs reaching the inner ear and CNS as a function of the starting dose and administration site (i.e., IP vs. IB). Direct measurements of drug levels in these compartments, however, will require perilymphatic and CSF sampling which are beyond the scope of the current study. However, we do have some insight into this relationship for a number of other substances which generally reach higher levels with middle ear applications than with systemic administration. With the IV/IP routes, perilymphatic levels of fluorescein, TMPA, salicylate, and IEM-1460 were 0.05–1.4% of the administered dose (Boettcher et al., 1990; Inamura and Salt, 1992; Hirose et al., 2014; Salt et al., 2018a; Walia et al., 2021). With round window application, the percentage of gentamicin, Dex-P, TMPA, and fluorescein found in the perilymph are typically higher ranging from 1 to 6% (Plontke et al., 2008; Mikulec et al., 2009; Salt et al., 2018a). Since blocking times are a function of drug levels reaching efferent synapses, it is important to compare similar final perilymphatic concentrations with different administration routes. For a 20 g mouse in our Gly and Msc experiments, we used the same stock solution (0.2 mg/ml), but different delivery volumes for IP (0.2 ml for 2 mg/kg) and IB (0.03 ml) to arrive at starting drug amounts of 40 and 6 μg, respectively. Assuming a ∼1 and 5% differential perilymphatic access among the IP and IB routes, estimates of final drug amounts reaching efferent synapses would be similar (i.e., 0.4 μg for IP and 0.3 μg for IB). For IC glycopyrrolate, assuming all of the drug administered (1 μl of 0.2 mg/ml stock solution) makes it into the perilymph, a final drug amount of 0.2 μg is within the same range. Future direct measurements of drug levels in the perilymph are needed to confirm if this relationship holds for glycopyrrolate and methylscopolamine as well as the other cholinergic efferent drugs. In accordance with existing literature (Domino and Corssen, 1967; Proakis and Harris, 1978; Freedman et al., 1989; Callegari et al., 2011; Wala et al., 2012; Walbaum, 2017; Chabicovsky et al., 2019), we postulate that these cholinergic drugs exhibit poor BBB penetration, but measurements of CSF drug levels in mice will also be important in determining to what extent, if any, these drugs have access to the CNS in our preparation.

### Delineating Peripheral Versus Central Actions of mAChR Antagonist

For glycopyrrolate and methscopolamine, we are relying on literature in multiple species and preparations using a variety of biochemical, electrophysiological, and behavioral assays to document that these drugs have little access across the BBB. But, direct measurements of drug levels in blood, perilymph, and CSF in our mouse preparation would be more reassuring. We know that the effectiveness of mAChR and nAChR antagonists to block efferent-mediated slow and fast excitation, respectively, occurs in the vestibular periphery and not as a function of changing the sensitivity of central efferent neurons to efferent stimulation. We know this because efferent-mediated fast excitation remains unchanged during mAChR blockade and efferent-mediated slow excitation remains unchanged during nAChR blockade. However, some mAChR blockers systematically affect background discharge and we have routinely, as a matter of transparency, described this phenomenon in our mouse efferent work.

While the effects on baseline discharge could be attributed to central effects with certain mAChR antagonists, we have not identified the specific target for these effects nor determined that similar mechanisms are involved with each mAChR antagonist. Schneider et al. (2021) demonstrated that the mAChR antagonists atropine and scopolamine, which do enter the CNS, affected background discharge when administered through the IP route but not the IB route suggesting that other central and/or peripheral mAChR targets may underlie this effect. However, in the current study, both IP and IB glycopyrrolate significantly decreased background discharge suggesting that the effects are peripheral. This can be contrasted with IP and IB methscopolamine where the effects on afferent background rates are neither consistent nor as pronounced, suggesting that perhaps differences in chemical structures between these two charged mAChR antagonists may permit glycopyrrolate, but not methscopolamine, to modify afferent discharge. Further exploration of the dose response relationship for these different mAChR antagonists may find doses where the effect on efferent-mediated slow excitation and background discharge can be easily separated.

The effect of mAChR antagonists on baseline discharge might be interpreted as a role for efferent tone, which would be consistent with recent data indicating that optothermal inhibition of vestibular efferent neurons in mice gives rise to decreased spontaneous activity in vestibular afferents (Raghu et al., 2019). Such efferent tone, if present in our anesthetized preparation, might result in the release of efferent neurotransmitters (e.g., ACh) that activate mAChRs and/or other peripheral vestibular efferent mechanisms to augment afferent discharge under control conditions. Subsequent blockade of central mAChRs could inhibit neurons in the vestibular nuclei, reticular formation, and/or *e* group to give rise to a decrease in efferent input at the end organ level that ultimately reduces afferent firing (Metts et al., 2006; Soto and Vega, 2010; Idoux et al., 2018). This scenario is consistent with our observations with IP atropine and IP scopolamine, and could be extended to IP and IB glycopyrrolate provided that appreciable amounts of drug, in both cases, reached the brainstem. In turn, IC glycopyrrolate, IP methscopolamine, and IB methscopolamine fail to breach the CNS and therefore do not impact the supposed efferent tone. The challenge with this interpretation is the difficulties in reconciling how IB administration of scopolamine and atropine do not target those same central mechanisms. Along the same lines, activation of the mAChRs that give rise to efferent-mediated slow excitation of vestibular afferents likely does not underlie the decreases in afferent firing seen with mAChR antagonists given the lack of correspondence between these two effects with different drugs and different routes. While previous sectioning experiments suggest that there is little efferent tone or basal activity in our preparation (Schneider et al., 2021), the effects of different anesthetic regimens (i.e., ketamine/xylazine vs. urethane/xylazine) on efferent tone cannot be ruled out as well as any interactions that may exist between each anesthetic cocktail and the different mAChR antagonists.

Alternatively, it is entirely conceivable that electrical stimulation of efferent neurons in our preparation could produce other long-term changes (over 10 s of minutes) in afferent firing, in addition to efferent-mediated slow excitation, that are both sensitive to mAChR blockade. This could all happen in the periphery without involving central efferent circuitry. We know from the literature, that there is also evidence for mAChRs on type II hair cells and vestibular supporting cells (Liu and Wangemann, 1998; Derbenev et al., 2005; Li et al., 2007; Li and Correia, 2011), whose activation could give rise to slower changes in afferent discharge rates. Access time to these receptors may lag behind those needed to block mAChRs on the afferent and thus may contribute to the failure to see background changes when the drugs are delivered quickly to the inner ear like IB scopolamine, IB atropine, and IC glycopyrrolate. When access times are longer, however, blockade of both groups of mAChRs will overlap as is the case with IP scopolamine and IP atropine (Schneider et al., 2021) as well as both IP and IB glycopyrrolate (this study). Longer incubation times with different routes might be helpful here. However, the lack of a consistent effect of methscopolamine on baseline, with either route, is hard to reconcile with such timing differences. The observations that IP and IB methscopolamine do not have significant effects on baseline firing could suggest that specific properties of methscopolamine (e.g., chemical structure, specificity, potency, etc.) may account for the differences. Binding kinetic parameters do vary among the different mAChR antagonists (Riddy et al., 2015). Currently, there is no single or unifying conclusion that satisfactorily explains our data collectively, suggesting that multiple mechanisms are likely involved.

### Drug Movement Between Ears

Another interesting outcome of this study was the observation that the contralateral middle ear application of glycopyrrolate and methscopolamine eventually reached the ipsilateral ear. A number of inner ear studies have reported similar observations with a number of different substances (Bath et al., 1999; Stöver et al., 2000; Landegger et al., 2017; Salt et al., 2018a; Lee et al., 2020; Lentz et al., 2020), but the current study, for the first time, tried to monitor the time course of drug communication between ears in mice. While the mechanism by which mAChR antagonists gain access to the ipsilateral ear in our preparation is unknown, possible routes from the contralateral to the ipsilateral ear include a perilymph to vascular route, perilymph to CSF route via the cochlear aqueduct, lymphatic pathways, and/or the eustachian tube (Salt and Hirose, 2018; Talaei et al., 2019; Lee et al., 2020). Fluorescent gentamicin, after injection into the perilymph of the posterior semicircular canal in mice, makes its way out into the systemic circulation in short as 1 h or less (Talaei et al., 2019). The time course for movement of contralateral glycopyrrolate and methscopolamine to the ipsilateral ear overlaps that of the fluorescent gentamicin suggesting similar mechanisms for both. Drug movement between ears may complicate the characterization of drug effects following local application to the ear, particularly with those drugs that can enter the CNS upon systemic redistribution. Further insight into these processes might be had after determining whether contralateral IC injection of glycopyrrolate also blocks efferent-mediated slow excitation in the ipsilateral ear. This approach could be used to exclude the eustachian tube route. Contralateral IC injection of the charged α9α10nAChR antagonists might also differentiate between a perilymph to systemic circulation route from a perilymph to CSF route.

### Significance of Glycopyrrolate and Methscopolamine in Understanding Efferent Vestibular System Function

It has been long recognized that mAChR antagonists like scopolamine are effective in alleviating motion sickness (Yates et al., 1998; Renner et al., 2005; Golding, 2016). The general consensus has been that scopolamine’s effectiveness is attributed to mAChR blockade in central vestibular circuitry (Soto and Vega, 2010; Idoux et al., 2018). But evidence regarding efferent activation of mAChRs on vestibular afferents and its sensitivity to mAChR antagonists, now including glycopyrrolate and methscopolamine, suggest we include mAChRs in the vestibular periphery as potential contributors (Weerts et al., 2015; Holt et al., 2017; Ramakrishna et al., 2020; Schneider et al., 2021). While differences in the effectiveness of peripherally and centrally-active mAChR antagonists in alleviating motion sickness are mixed (Kirsten and Schoener, 1975; Uijdehaage et al., 1993; Hasler et al., 1995; Lang et al., 1999; Spinks and Wasiak, 2011; Qi et al., 2019), glycopyrrolate has been utilized as a vestibular suppressant in Meniere’s patients and for treating vertigo after cochleostomy during cochlear implants (Storper et al., 1998; Chakrabarty et al., 2011). Collectively, these data suggest that pharmacological targeting of mAChRs in the vestibular periphery may be of some utility in treating motion sickness, and that glycopyrrolate and methscopolamine could be used to distinguish the role of mAChRs in peripheral and central vestibular circuitry. Revisiting the effect of motion sickness on VOR efficacy (Idoux et al., 2018), and assessing susceptibility to charged mAChR antagonists could be instructive in this regard.

## Data Availability Statement

The raw data supporting the conclusions of this article will be made available by the authors, without undue reservation.

## Ethics Statement

The animal study was reviewed and approved by University Committee for Animal Resources (UCAR) at the University of Rochester Medical Center (URMC).

## Author Contributions

CL, PC, and JH contributed to study concept and design. CL, AS, AW, KH, and JH were instrumental in the acquisition of electrophysiological and pharmacological data. CL, JH, and KH were involved in the analysis and interpretation of the data. The manuscript was written and revised by all authors.

## Conflict of Interest

The authors declare that the research was conducted in the absence of any commercial or financial relationships that could be construed as a potential conflict of interest.

## Publisher’s Note

All claims expressed in this article are solely those of the authors and do not necessarily represent those of their affiliated organizations, or those of the publisher, the editors and the reviewers. Any product that may be evaluated in this article, or claim that may be made by its manufacturer, is not guaranteed or endorsed by the publisher.

## Footnotes

1
http://www.swissadme.ch
